# Supplementary material for: Brain-wide representations of prior information in mouse decision-making
Source: Nature. 2025 Sep 3;645(8079):192–200. doi: 10.1038/s41586-025-09226-1 (PMC12408363; doi:10.1038/s41586-025-09226-1)
Supplement: Supplementary file 1 — Behavioural models, hierarchical model and details of the IBL consortium. [file 41586_2025_9226_MOESM1_ESM.pdf]

nature portfolio

https://doi.org/10.1038/s41586-025-09226-1

Supplementary information

Brain-wide representations of prior information in mouse decision-making

In the format provided by the authors and unedited

1 Behavioural models

The behavioural models section is divided into four subsections:

Prior modelling: describes the estimation of the prior belief as to where the stimulus is going to appear on the next trial.

Evidence model: describes how the evidence presented on the screen (the stimulus, in non-zero-contrast trials), is processed by the sensory areas

Decision policy: describes how the prior and the evidence are combined to lead to a choice

Full likelihood: makes explicit the full likelihood which is used to fit the behavioural model to the mouse's choices.

1.1 Prior modelling

Here, we describe the three different models of the construction of the prior belief as to where the stimulus will appear on the next trial (in the mouse's internal model).

1.1.1 Bayes Optimal: prior from the generative Bayesian model of the task

Let l_t be the current length of the current block. Let b_t be the current block label ($b_t = 0$ indicates unbiased, $b_t = -1$ indicates left-biased and $b_t = 1$ indicates right-biased). Let s_t be the stimulus side ($s_t = -1$ indicates stimulus on left side, $s_t = 1$ indicates stimulus on right side).

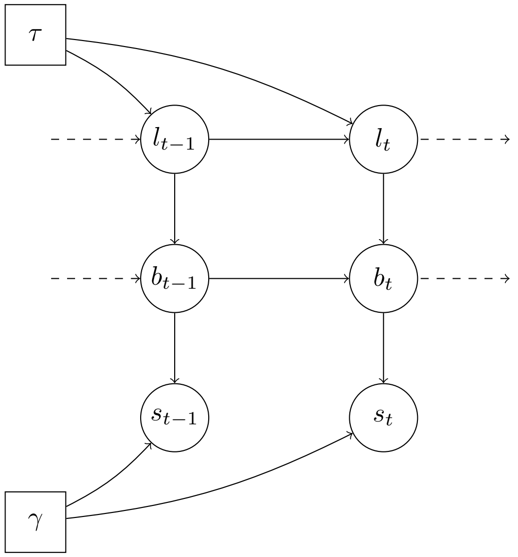

with $\tau = 60$ the scale of the exponential distribution prior over block lengths. Block length has two additional parameters not shown here, the minimum block size (20 trials) and the maximum block size (100 trials). $\gamma = 0.8$ defines the probability that the stimulus appears on a side given the block.

Analytical equations

Dynamics over the current length of the current blocks:

$$l_t | l_{t-1} = \begin{cases} 1 & \text{with probability } H_\tau(l_{t-1}) \\ l_{t-1} + 1 & \text{with probability } 1 - H_\tau(l_{t-1}) \end{cases}$$

with $l_1 = 1$, $H_\tau(n) = h(n)/\sum_{100 \geq n' \geq n}[h(n')]$ and $h(n) = \exp(-n/\tau) \cdot [20 \leq n \leq 100]$

Dynamics over blocks:

$$b_t | b_{t-1}, l_t = \begin{cases} b_{t-1} & \text{if } l_t > 1 \\ -b_{t-1} & \text{if } l_t = 1 \text{ and } b_{t-1} \neq 0 \\ U(\{-1, 1\}) & \text{if } l_t = 1 \text{ and } b_{t-1} = 0 \end{cases}$$

With $b_1 = 0$.

Dynamics over the stimulus side (left or right):

$$s_t | b_t = \begin{cases} U(\{-1, 1\}) & \text{if } b_t = 0 \\ b_t & \text{with probability } \gamma \text{ if } b_t \neq 0 \\ -b_t & \text{with probability } 1 - \gamma \text{ if } b_t \neq 0 \end{cases}$$

In other words: $p(s_t|b_t) = (1/2)^{b_t=0} \cdot \gamma^{(b_t \neq 0)\cdot(s_t = b_t)} \cdot (1-\gamma)^{(b_t \neq 0)\cdot(s_t \neq b_t)}$

Importantly, this generative model ignores the structure of the first 90 unbiased trials. It assumes that the first block is unbiased but doesn't force the 90 first trials to be unbiased - the unbiased first block is assumed to follow the same statistics as any other block. Indeed, a Bayesian model selection comparing two Bayesian models revealed that, relative to the model that enforces the first 90 trials to be unbiased, the variant that treats those trials like any other block is overwhelmingly preferred, with an exceedance probability exceeding0.999.

Inference To perform inference, meaning to obtain the priors $\pi_t = p\left(s_t = 1 | s_{1:(t-1)}\right)$, we apply the likelihood recursion algorithm [1].

Let us define the forward variables

$$\begin{aligned} g_t(l_t, b_t) &= p(l_t, b_t, s_{1:(t-1)}) \\ h_t(l_t, b_t) &= p(l_t, b_t, s_{1:t}) \end{aligned}$$

as the joint likelihoods of l_t, b_t and $s_{1:(t-1)}$ or $s_{1:t}$, respectively.

The likelihood recursion (with conditional independencies, see graphical model) gives:

$$\begin{aligned} h_t(l_t, b_t) &= \sum_{l_{t-1}, b_{t-1}} p(s_t, s_{1:(t-1)}, l_t, b_t, l_{t-1}, b_{t-1}) \\ &= \sum_{l_{t-1}, b_{t-1}} p(s_{1:(t-1)}, l_{t-1}, b_{t-1}|) \cdot p(l_t|l_{t-1}) \cdot p(b_t|b_{t-1}, l_t) \cdot p(s_t|b_t) \\ &= p(s_t|b_t) \cdot \sum_{l_{t-1}, b_{t-1}} h_{t-1}(l_{t-1}, b_{t-1}) \cdot p(l_t|l_{t-1}) \cdot p(b_t|b_{t-1}, l_t) \\ &= p(s_t|b_t) \cdot g_t(l_t, b_t) \end{aligned}$$

with,

$$g_t(l_t, b_t) = \sum_{l_{t-1}, b_{t-1}} h_{t-1}(l_{t-1}, b_{t-1}) \cdot p(l_t|l_{t-1}) \cdot p(b_t|b_{t-1}, l_t)$$

Obtaining the *prior* $\pi_t = p\left(s_t = 1 | s_{1:(t-1)}\right)$ is now straightforward:

$$\pi_t = 0.5 \cdot p\left(b_t = 0 | s_{1:(t-1)}\right) + \gamma \cdot p\left(b_t = 1 | s_{1:(t-1)}\right) + (1-\gamma) \cdot p\left(b_t = -1 | s_{1:(t-1)}\right)$$

with

$$p\left(b_t = k | s_{1:(t-1)}\right) = \frac{\sum_{l_t} g_t\left(l_t, b_t = k\right)}{\sum_{l_t, b_t} g_t\left(l_t, b_t\right)}$$

Algorithm Let T be the total number of trials, and with the notation of the previous paragraph.

Algorithm 1: Inference of the generative model

Result: priors $\pi_t = p(s_t = 1|s_{1:(t-1)})$ at every trials t
initialize $g_1(l_1, b_1) = p(l_1, b_1)$ for all (l_1, b_1)
for $t = 1$; $t \leq T$; $t++$ **do**
  for *all* (l_t, b_t) **do**
    if $t \geq 2$ **then**
      $g_t(l_t, b_t) = \sum_{l_{t-1}, b_{t-1}} h_{t-1}(l_{t-1}, b_{t-1}) \cdot p(l_t|l_{t-1}) \cdot p(b_t|b_{t-1}, l_t)$
    end
    $h_t(l_t, b_t) = p(s_t|b_t) \cdot g_t(l_t, b_t)$
  end
  $\pi_t = 0.5 \cdot p\left(b_t = 0|s_{1:(t-1)}\right) + \gamma \cdot p\left(b_t = 1|s_{1:(t-1)}\right) + (1-\gamma) \cdot p\left(b_t = -1|s_{1:(t-1)}\right)$
  with, $p\left(b_t = k|s_{1:(t-1)}\right) = \frac{\sum_{l_t} g_t(l_t, b_t = k)}{\sum_{l_t, b_t} g_t(l_t, b_t)}$
end

Establishing the equivalence between the HMM formulation and the semi-hidden Markov formulation of the task We verify here that the block length generated with the previously described Markovian process follows the correct truncated exponential distribution.
Let us define the random variable X_m as the length of block number m. We can write X_m as the random variable $(l_t, l_{t+1} = 1)$, the length of the block that ends at trial t.
Do we have $P(X_m = k) = \left(\frac{e^{-k/\tau}}{\sum_{n=20}^{100} e^{-n/\tau}}\right)$, for all $k \in [20, 100]$?

$$\begin{aligned}
P(X_m = k) &= P(l_t = k,\ l_{t+1} = 1) \\
&= \prod_{i=20}^{k-1} (1 - H_\tau(i)) \cdot H_\tau(k) \\
&= \left(\frac{e^{-k/\tau}}{\sum_{n=k}^{100} e^{-n/\tau}}\right) \cdot \prod_{i=20}^{k-1} \left(1 - \frac{e^{-i/\tau}}{\sum_{n=i}^{100} e^{-n/\tau}}\right) \propto \left(\frac{e^{-k/\tau}}{\sum_{n=k}^{100} e^{-n/\tau}}\right) \cdot \prod_{i=20}^{k-1} \left(\frac{\sum_{n=i+1}^{100} e^{-n/\tau}}{\sum_{n=i}^{100} e^{-n/\tau}}\right) \\
&= \left(\frac{e^{-k/\tau}}{\sum_{n=20}^{100} e^{-n/\tau}}\right) \cdot \left(\frac{\sum_{n=21}^{100} e^{-n/\tau}}{\sum_{n=21}^{100} e^{-n/\tau}} \ \cdots \ \frac{\sum_{n=k}^{100} e^{-n/\tau}}{\sum_{n=k}^{100} e^{-n/\tau}}\right) \\
&= \left(\frac{e^{-k/\tau}}{\sum_{n=20}^{100} e^{-n/\tau}}\right)
\end{aligned}$$

which concludes the equivalence

1.1.2 Stimulus Kernel: heuristic prior based on stimulus side integration

A second prior model consists in integrating over previous stimulus sides with an exponentially decaying kernel.

$$\pi_t | \pi_{t-1}, s_{t-1}, \alpha = (1 - \alpha) \cdot \pi_{t-1} + \alpha \cdot (s_{t-1} > 0)$$

where $s_{t-1} \in \{-1, 1\}$ is the stimulus side presented on trial $t - 1$ and α is the learning rate.

Given all stimuli observed by the mouse $s_{1:T}$, and the learning rate α, obtaining the heuristic prior π_t is trivial.

To take into account the possibility of positivity and confirmation biases, we considered an alternative

stimulus kernel model with asymmetrical learning rates. For this alternative, we track two variables, $\widehat{\pi}_t^{left}$ and $\widehat{\pi}_t^{right}$, which are unnormalized heuristic priors associated with the left and right stimulus side. The updating rule assumes now the existence of 4 learning rates.

- α_+^c : the learning rate associated with the chosen side if the chosen side was rewarded
- α_+^u : the learning rate associated with the unchosen side if the chosen side was unrewarded
- α_-^u : the learning rate associated with the unchosen side if the chosen side was rewarded
- α_-^c : the learning rate associated with the chosen side if the chosen side was unrewarded

The updating rule for this stimulus-side integration alternative is the following:

$$\begin{aligned}\widehat{\pi}_t^{left} &= \widehat{\pi}_{t-1}^{left} + \alpha_t^{left} \cdot \Big((s_{t-1} < 0) - \widehat{\pi}_{t-1}^{left}\Big)\\ \widehat{\pi}_t^{right} &= \widehat{\pi}_{t-1}^{right} + \alpha_t^{right} \cdot \Big((s_{t-1} > 0) - \widehat{\pi}_{t-1}^{right}\Big)\end{aligned}$$

with $\alpha_t^{left} = (a_{t-1} = -1)\left[\alpha_+^c \cdot (s_{t-1} = a_{t-1}) + \alpha_-^c \cdot (s_{t-1} \neq a_{t-1})\right] + (a_{t-1} = 1)\left[\alpha_+^u \cdot (s_{t-1} = a_{t-1}) + \alpha_-^u \cdot (s_{t-1} \neq a_{t-1})\right]$ and equivalently for α_t^{right}.

The prior π_t is then set according to:

$$\pi_t = \frac{\widehat{\pi}_t^{right}}{\widehat{\pi}_t^{left} + \widehat{\pi}_t^{right}}$$

1.1.3 Action kernel: heuristic prior based on action integration

A third prior model consists in integrating previous actions with an exponentially decaying kernel.

$$\pi_t | \pi_{t-1}, a_{t-1}, \alpha = (1 - \alpha) \cdot \pi_{t-1} + \alpha \cdot (a_{t-1} > 0)$$

with $a_{t-1} \in \{-1, 1\}$ the action performed by the mouse at time $t - 1$ and α the learning rate.

Given all actions performed by the mouse $a_{1:T}$ and the learning rate α, obtaining the heuristic prior π_t is trivial.

1.2 Evidence model

We present here a generative model of the mouse's internal estimate of the stimulus contrast. We will assume here that the stimulus side is inferred in the optimal way given a noisy stimulus contrast and a subjective prior.

Let s_t be the stimulus side, c_t be the exact stimulus contrast at trial t, and m_t its noisy version. We consider the mouse to have a single noisy representation m_t of c_t which it uses to make choices.

1.2.1 Graphical representation

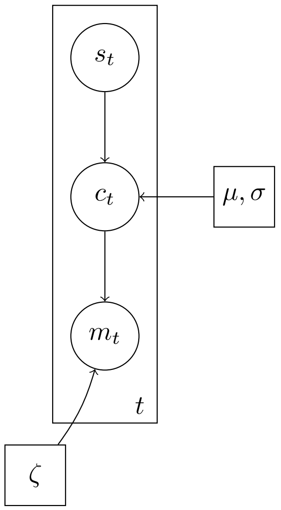

1.2.2 Analytical equations

The noise structure assumed is to be doubly Gaussian:

$$\begin{aligned} p\left(c_t | s_t = 1\right) &\propto f\left(c_t; \mu, \sigma\right) \cdot \mathbb{1}[c_t \geq 0] \\ p\left(c_t | s_t = -1\right) &\propto f\left(c_t; \mu, \sigma\right) \cdot \mathbb{1}[c_t \leq 0] \\ m_t \mid c_t, \zeta &\sim \mathcal{N}\left(c_t, \zeta\right) \end{aligned}$$

with f the Gaussian probability density function. We will assume that

$$\begin{aligned} \mu &= 0 \\ \sigma^2 &= \sum_{c \in \{-1, -0.25, -0.125, -0.0625, 0, 0.625, 0.125, 0.25, 1\}} 1/9 \cdot c^2 = 0.24 \end{aligned}$$

We make this assumption of a doubly Gaussian noise structure to make the inference tractable.

1.2.3 Inference

With s_t known, to compute the conditional probability of the measurement m_t given the side s_t, we need to marginalize over the contrast c_t. This gives;

$$\begin{aligned} p(m_t \mid s_t = 1, \zeta) &= \int_{c_t} p(m_t, c_t \mid s_t = 1, \zeta) dc_t = \int_{c_t} p(c_t \mid s_t = 1) \cdot p(m_t \mid c_t, \zeta) dc_t \\ &= \int_{c_t > 0} f(c_t; \mu, \sigma) \cdot f(m_t \,; c_t, \zeta) dc_t = \int_{c_t > 0} \psi_t \cdot f(c_t \,; \mu_t^*, \sigma_t^*) dc = \psi_t \cdot \Phi\left(\frac{\mu_t^*}{\sigma^*}\right) \end{aligned}$$

with f and Φ the probability density function and cumulative density function of a Gaussian distribution and:

$$\begin{aligned}
\psi_t &= f\left(\mu;\ m_t,\ \sigma^2+\zeta^2\right) \\
(\sigma^*)^2 &= \frac{1}{\frac{1}{\sigma^2}+\frac{1}{\zeta^2}} \\
\mu_t^* &= (\sigma^*)^2 \cdot \left(\frac{\mu}{\sigma^2}+\frac{m_t}{\zeta^2}\right) = \frac{\sigma^{-2}\cdot\mu+\zeta^{-2}\cdot m_t}{\sigma^{-2}+\zeta^{-2}} = \frac{\zeta^{-2}\cdot m_t}{\sigma^{-2}+\zeta^{-2}}
\end{aligned}$$

1.3 Decision policy of the mouse

1.3.1 Posterior over s_t

We assume that the animal computes the posterior $p\left(s_t|m_t, s_{1:(t-1)}\right)$ following Bayes rule, i.e., by combining likelihood and subjective prior.

$$p\left(s_t = 1|m_t, s_{1:(t-1)}\right) \propto \psi_t \cdot \pi_t \cdot \Phi\left(\frac{\mu_t^*}{\sigma^*}\right)$$
$$p\left(s_t = -1|m_t, s_{1:(t-1)}\right) \propto \psi_t \cdot (1-\pi_t) \cdot \left(1 - \Phi\left(\frac{\mu_t^*}{\sigma^*}\right)\right)$$

1.3.2 Optimal Policy with lapse rates

If the animal would seek to maximize the probability of reward, then it should pick the side s_t with a higher posterior probability given its internal measurement m_t. This corresponds to the decision rule:

$$\psi_t \cdot \pi_t \cdot \Phi\left(\frac{\mu_t^*}{\sigma^*}\right) < \psi_t \cdot (1-\pi_t) \cdot \left(1 - \Phi\left(\frac{\mu_t^*}{\sigma^*}\right)\right) \implies \text{"choose left"};\ a_t = -1$$
$$\psi_t \cdot \pi_t \cdot \Phi\left(\frac{\mu_t^*}{\sigma^*}\right) > \psi_t \cdot (1-\pi_t) \cdot \left(1 - \Phi\left(\frac{\mu_t^*}{\sigma^*}\right)\right) \implies \text{"choose right"};\ a_t = 1$$
$$\psi_t \cdot \pi_t \cdot \Phi\left(\frac{\mu_t^*}{\sigma^*}\right) = \psi_t \cdot (1-\pi_t) \cdot \left(1 - \Phi\left(\frac{\mu_t^*}{\sigma^*}\right)\right) \implies \text{"choose randomly"}$$

After simplification

$$\mu_t^* < \sigma^* \cdot \Phi^{-1}(1-\pi_t) \implies \text{"choose left"};\ a_t = -1$$
$$\mu_t^* > \sigma^* \cdot \Phi^{-1}(1-\pi_t) \implies \text{"choose right"};\ a_t = 1$$
$$\mu_t^* = \sigma^* \cdot \Phi^{-1}(1-\pi_t) \implies \text{"choose randomly"}$$

where $\Phi^{-1}(.)$ denotes the inverse cumulative normal distribution. Thus, the optimal decision rule corresponds to a simple threshold, where the animal chooses "right" whenever its corrected internal measurement μ^* exceeds $\sigma^*\Phi^{-1}(1-\pi_t)$ and otherwise chooses "left" (with a random choice in the equality case). When the prior is uniform, $\pi_t = 0.5$, then this simplifies to choosing according to the sign of m_t.

When adding a lapse rate ϵ_t, this decision rule can be expressed in full as

$$a_t = \text{sign}\left(\mu_t^* - \sigma^*\Phi^{-1}(1-\pi_t)\right) \qquad \text{with probability } 1-\epsilon_t$$
$$\sim \mathcal{U}(\{-1; 1\}) \qquad \text{with probability } \epsilon_t$$

with, $\epsilon_t = \epsilon^+ \cdot \mathbb{1}[c_t > 0] + \epsilon^- \cdot \mathbb{1}[c_t < 0] + (\epsilon^+ + \epsilon^-) \cdot 0.5 \cdot \mathbb{1}[c_t = 0]$, $\sigma^2 = 0.24$ and

$$(\sigma^*)^2 = \frac{1}{\frac{1}{\sigma^2} + \frac{1}{\zeta^2}} \qquad \mu_t^* = \frac{\zeta^{-2} \cdot m_t}{\sigma^{-2} + \zeta^{-2}}$$

To take into account the equality case, we assume that sign(0) = (-1 with probability 0.5 else 1)
We assume here contrast-side dependent lapse rates, meaning that the lapse rates will depend on whether the contrast was on the left or right side. Bayesian model comparison indicated that letting lapse rates differ by stimulus side provides a better account of behaviour compared with a single lapse rate, yielding an exceedance probability greater than 0.999.. These contrast-side dependent lapse rates would typically occur if mice pay more attention to one side than to the other.

Additionally, two heuristics were incorporated into the decision policy for the Bayes optimal and stimulus kernel models to account for potential autocorrelations in actions. The first is a repetition bias was implemented as follows:

$$
\begin{aligned}
a_t &= \operatorname{sign}\left(\mu_t^* - \sigma^* \Phi^{-1}(1-\pi_t)\right) && \text{with probability } 1-\epsilon_t-\eta \\
&= a_{t-1} && \text{with probability } \eta \\
&\sim \mathcal{U}(\{-1;1\}) && \text{with probability } \epsilon_t
\end{aligned}
$$

With η the repetition bias parameter.

The second is a multi-step repetition bias (also referred to as choice trace) was implemented as follows:

$$
\begin{aligned}
a_t &= \operatorname{sign}\left(\mu_t^* - \sigma^* \Phi^{-1}(1-\pi_t)\right) && \text{with probability } 1-\epsilon_t-\eta \\
&= F_\lambda(a_{1:(t-1)}) && \text{with probability } \eta \\
&\sim \mathcal{U}(\{-1;1\}) && \text{with probability } \epsilon_t
\end{aligned}
$$

In this case, η denotes the multi-step repetition bias parameter and F_λ is a function that applies an exponentially decaying average over previous actions, with λ controlling the rate of temporal decay. Again, to take into account the equality case, we assume that sign(0) = (-1 with probability 0.5 else 1)

1.4 Full likelihood

The Markovian process $\{\pi_t,\ t \in [1,T]\}$ corresponds to the priors defined in subsection 1.1. θ are the parameters of the prior model. $\theta = \emptyset$ if the Bayesian model is considered and $\theta = \{\alpha\}$ for the heuristic models (and $\theta = \left\{\alpha_+^c, \alpha_+^u, \alpha_-^c, \alpha_-^u\right\}$ for the stimulus kernel with choice- and outcome-dependent learning rates).

Let us now write the likelihood of the mouse's action a_t marginalizing out the noisy contrast measurements m_t. We write down the equations in the case of a decision policy with lapse rates but the calculations are very similar when additionally considering a repetition bias.

$$
\begin{aligned}
p(a_t=1|c_t,\pi_t,\zeta,\theta,\epsilon_+,\epsilon_-) &= \int_{m_t} p(a_t=1,m_t|c_t,\pi_t)dm_t = \int_{m_t} p(m_t|c_t,\pi_t)\cdot p(a_t=1|m_t,\pi_t)dm_t \\
&= \int_{m_t} f\left(m_t;c_t,\zeta\right)\cdot \mathbb{1}\left[\mu_t^* > \sigma^*\Phi^{-1}(1-\pi_t)\right] dm_t \cdot (1-\epsilon_t) + \epsilon_t/2
\end{aligned}
$$

with $\mu_t^* = \frac{\zeta^{-2}}{\sigma^{-2}+\zeta^{-2}} \cdot m_t$, $\epsilon_t = \epsilon^+ \cdot \mathbb{1}[c_t > 0] + \epsilon^- \cdot \mathbb{1}[c_t > 0] + (\epsilon^+ + \epsilon^-) \cdot 0.5 \cdot \mathbb{1}[c_t = 0]$ and f the Gaussian pdf.

For the first term:

$$
\begin{aligned}
\int_{m_t} f\left(m_t;c_t,\zeta\right)\cdot \mathbb{1}\left[m_t > \left(\frac{\zeta^2}{\sigma^2}+1\right)\sigma^*\Phi^{-1}(1-\pi_t)\right] dm_t &= \int_{\left(\frac{\zeta^2}{\sigma^2}+1\right)\sigma^*\Phi^{-1}(1-\pi_t)}^{+\infty} f\left(m_t;c_t,\zeta\right)dm_t \\
&= \int_0^{+\infty} f\left(m_t; c_t - \left(\frac{\zeta^2}{\sigma^2}+1\right)\sigma^*\Phi^{-1}(1-\pi_t), \zeta\right) dm_t \\
&= \Phi\left(\frac{c_t}{\zeta} - \left(\frac{\zeta^2}{\sigma^2}+1\right)\frac{\sigma^*}{\zeta}\Phi^{-1}(1-\pi_t)\right)
\end{aligned}
$$

Thus:

$$
p(a_t=1|c_t,\pi_t,\zeta,\theta,\epsilon_+,\epsilon_-) = \Phi\left(\frac{c_t}{\zeta} - \left(\frac{\zeta^2}{\sigma^2}+1\right)\frac{\sigma^*}{\zeta}\Phi^{-1}(1-\pi_t)\right)\cdot(1-\epsilon_t) + \frac{\epsilon_t}{2}
$$

Given all actions of a mouse on a session $a_{1:T}$ ($a_t \in \{-1, 1\}$) and the corresponding contrasts $c_{1:T}$ ($c_t \in \{-1, -0.25, -0.125, -0.0625, 0, 0.0625, 0.125, 0.25, 1\}$), we obtain the loglikelihood:

$$\begin{aligned}\log\ p\left(a_{1:T}|c_{1:T}, \pi_{1:T}, \zeta, \theta, \epsilon_+, \epsilon_-\right) &= \sum_{t=1}^{T} \log\ p\left(a_t|c_t, \pi_t, \zeta, \theta, \epsilon_+, \epsilon_-\right) \\ &= \sum_{t=1}^{T} \mathbb{1}[a_t = 1] \cdot \log\ \rho_t + \mathbb{1}[a_t = -1] \log\ (1 - \rho_t)\end{aligned}$$

with

$$\begin{aligned}\rho_t &= \Phi\left(\frac{c_t}{\zeta} - \left(\frac{\zeta^2}{\sigma^2} + 1\right)\frac{\sigma^*}{\zeta}\Phi^{-1}\left(1 - \pi_t\right)\right) \cdot (1 - \epsilon_t) + \frac{\epsilon_t}{2} \\ \epsilon_t &= \epsilon^+ \cdot \mathbb{1}[c_t > 0] + \epsilon^- \cdot \mathbb{1}[c_t > 0] + (\epsilon^+ + \epsilon^-) \cdot 0.5 \cdot \mathbb{1}[c_t = 0] \\ (\sigma^*)^2 &= \frac{1}{\frac{1}{\sigma^2} + \frac{1}{\zeta^2}}\end{aligned}$$

π_t is a function of θ. Inference in the generative model of mouse behaviour implies computing the posterior over the parameters θ, ϵ_+, ϵ_-, ζ.

This is done by implementing a random walk adaptive Metropolis-Hastings [2] [3]. The procedure is adaptive as it learns the covariance matrix of the random walk to obtain relevant samples and acceptance ratios (see articles for more information). Furthermore, 4 chains are run in parallel and the Gelman-Rubin factor is used to assess convergence. In terms of priors, we use uniform distributions ranging from 0 to 1 for both the learning rates and sensory noise, ζ. Furthermore, we set uniform priors between 0 and 0.5 for the lapse rates and repetition biases.

NB: For the Bayes optimal model, we do not perform inference over $\{\tau, \gamma\}$, we fix them to their "correct" values.

2 Hierarchical model

We designed a hierarchical model that accounts for two types of variability: within individual mice and within sessions for a given mouse. This model is employed to evaluate the correlation between neural and behavioral learning rates. It establishes session-level parameters that are derived from distributions at the mouse level, which in turn are based on distributions at the population level.

2.1 Model Specification

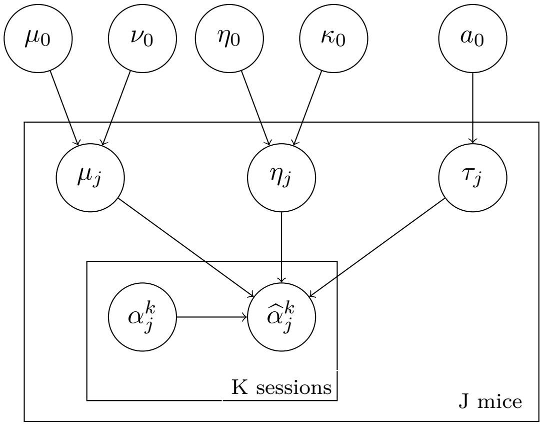

Linear Regression:

$$\widehat{\alpha}_j^k | \mu_j, \eta_j, \tau_j, \alpha_j^k \sim \mathcal{N}(\mu_j \cdot \alpha_j^k + \eta_j, \tau_j)$$

with $\widehat{\alpha}_j^k$ the learning rate (inverse decay constant) estimated from the neural activity and α_j^k the learning rate (inverse decay constant) estimated from behaviour. This linear regression is defined for each session k and mouse j. μ_j is the slope, η_j the intercept and τ_j the standard deviation of the linear regression defined at the mouse-level: these parameters are shared across all sessions for the same mouse.

Mouse-level parameters:

$$\mu_j | \mu_0, \nu_0 \sim \mathcal{N}(\mu_0, \nu_0)$$
$$\eta_j | \eta_0, \kappa_0 \sim \mathcal{N}(\eta_0, \kappa_0)$$
$$\tau_j \sim Exp(a_0)$$

The mouse-level slopes μ_j and intercepts η_j are assumed to follow Gaussian distributions and the standard deviations τ_j are assumed to follow an exponential distribution. These priors are defined with population-level parameters. μ_0 and ν_0 are the mean and standard deviation of the Gaussian prior over the mouse-level slopes μ_j. η_0 and κ_0 are the mean and standard deviation of the Gaussian prior over the mouse-level intercepts η_j. Lastly, a_0 is the parameter of the exponential prior over mouse-level standard deviations τ_j.

Population-level parameters:

$$\mu_0 \sim N(0, 1)$$
$$\nu_0 \sim Exp(0.1)$$
$$\eta_0 \sim N(0, 1)$$
$$\kappa_0 \sim Exp(0.1)$$
$$a_0 \sim Exp(0.1)$$

These last equations describe the priors over the population-level parameters μ_0, ν_0, η_0, κ_0 and a_0.

2.2 Inference

Inference is done with Metropolis-Hasting, running a chain for 50000 iterations and selecting the second half of samples to compute the posteriors.

The main random variables of interest are μ_0 and $\{\mu_j\}_j$, the slopes at the population level and at the mouse level. These are shown in extended data figure 15.

References

[1] Scott, S. L. (2002). Bayesian methods for hidden Markov models: Recursive computing in the 21st century. *Journal of the American statistical Association, 97(457)*, 337-351.

[2] Andrieu, C., & Thoms, J. (2008). A tutorial on adaptive MCMC. *Statistics and computing, 18*, 343-373.

[3] Baker, M. J. (2014). Adaptive Markov chain Monte Carlo sampling and estimation in Mata. *The Stata Journal, 14(3)*, 623-661.

IBL Consortium

First	Middle	Last	Current Affiliation
Larry		Abbot	Zuckerman Institute, Columbia University, New York, NY, USA
Luigi		Acerbi	Department of Basic Neuroscience, University of Geneva, Geneva, Switzerland
Valeria		Aguillon-Rodriguez	Cold Spring Harbor Laboratory, Cold Spring Harbor, NY, USA
Mandana		Ahmadi	Gatsby Computational Neuroscience Unit, University College London, London, UK
Jaweria		Amjad	Gatsby Computational Neuroscience Unit, University College London, London, UK
Dora		Angelaki	Center for Neural Science, New York University, New York, NY, USA
Jaime		Arlandis	Champalimaud Center for the Unknown, Lisboa, Portugal
Zoe	C	Ashwood	Princeton Neuroscience Institute, Princeton University, Princeton, NJ, USA
Kush		Banga	Institute of Neurology, University College London, London, UK
Hailey		Barrell	Department of Biological Structure, University of Washington, Seattle, WA, USA
Hannah	M	Bayer	Zuckerman Institute, Columbia University, New York, NY, USA
Brandon		Benson	Department of Applied Physics, Stanford University, Stanford, CA, USA
Julius		Benson	Center for Neural Science, New York University, New York, NY, USA
Jai		Bhagat	Institute of Neurology, University College London, London, UK
Dan		Birman	Department of Biological Structure, University of Washington, Seattle, WA, USA
Niccolò		Bonacchi	Champalimaud Center for the Unknown, Lisboa, Portugal
Kcenia		Bougrova	Champalimaud Center for the Unknown, Lisboa, Portugal
Julien		Boussard	Zuckerman Institute, Columbia University, New York, NY, USA
Sebastian	A	Bruijns	Max Planck Institute for Biological Cybernetics, Tübingen, Germany
Kelly	E	Buchanan	Zuckerman Institute, Columbia University, New York, NY, USA
Robert		Campbell	Sainsbury-Wellcome Centre, University College London, London, UK
Matteo		Carandini	Institute of Ophthalmology, University College London, London, United Kingdom
Joana	A	Catarino	Champalimaud Center for the Unknown, Lisboa, Portugal
Fanny		Cazettes	Champalimaud Center for the Unknown, Lisboa, Portugal
Gaelle	A	Chapuis	Department of Basic Neuroscience, University of Geneva, Geneva, Switzerland

Anne	K	Churchland	Department of Neurobiology, University of California, Los Angeles, CA, USA
Davide		Crombie	Champalimaud Center for the Unknown, Lisboa, Portugal
Yang		Dan	Department of Molecular and Cell Biology, University of California, Berkeley, CA, USA
Felicia		Davatolhagh	Department of Neurobiology, University of California, Los Angeles, CA, USA
Peter		Dayan	Max Planck Institute for Biological Cybernetics, Tübingen, Germany
Sophie		Denève	Département d'Études Cognitives, École Normale Supérieure, Paris, France
Eric	EJ	DeWitt	Champalimaud Center for the Unknown, Lisboa, Portugal
Tatiana		Engel	Princeton Neuroscience Institute, Princeton University, Princeton, NJ, USA
Michele		Fabbri	Zuckerman Institute, Columbia University, New York, NY, USA
Mayo		Faulkner	Institute of Neurology, University College London, London, UK
Robert		Fetcho	Princeton Neuroscience Institute, Princeton University, Princeton, NJ, USA
Ila		Fiete	Department of Brain and Cognitive Sciences, Massachusetts Institute of Technology, Cambridge, MA, USA
Charles		Findling	Department of Basic Neuroscience, University of Geneva, Geneva, Switzerland
Laura		Freitas-Silva	Champalimaud Center for the Unknown, Lisboa, Portugal
Surya		Ganguli	Department of Applied Physics, Stanford University, Stanford, CA, USA
Berk		Gercek	Department of Basic Neuroscience, University of Geneva, Geneva, Switzerland
Naureen		Ghani	Sainsbury-Wellcome Centre, University College London, London, UK
Ivan		Gordeliy	Département d'Études Cognitives, École Normale Supérieure, Paris, France
Laura	M	Haetzel	Princeton Neuroscience Institute, Princeton University, Princeton, NJ, USA
Kenneth	D	Harris	Institute of Neurology, University College London, London, UK
Michael		Häusser	Wolfson Institute of Biomedical Research, University College London, London, United Kingdom
Naoki		Hiratani	Gatsby Computational Neuroscience Unit, University College London, London, UK
Sonja		Hofer	Sainsbury-Wellcome Centre, University College London, London, UK
Fei		Hu	Department of Molecular and Cell Biology, University of California, Berkeley, CA, USA
Felix		Huber	Department of Basic Neuroscience, University of Geneva, Geneva, Switzerland
Julia	M	Huntenburg	Max Planck Institute for Biological Cybernetics, Tübingen, Germany

Cole		Hurwitz	Zuckerman Institute, Columbia University, New York, NY, USA
Anup		Khanal	Department of Neurobiology, University of California, Los Angeles, CA, USA
Christopher	S	Krasniak	Cold Spring Harbor Laboratory, Cold Spring Harbor, NY, USA; Watson School of Biological Sciences, Cold Spring Harbor, NY, USA
Sanjukta		Krishnagopal	Gatsby Computational Neuroscience Unit, University College London, London, UK
Michael		Krumin	Institute of Neurology, University College London, London, UK
Debottam		Kundu	Max Planck Institute for Biological Cybernetics, Tübingen, Germany
Agnès		Landemard	Institute of Ophthalmology, University College London, London, United Kingdom
Christopher		Langdon	Princeton Neuroscience Institute, Princeton University, Princeton, NJ, USA
Christopher		Langfield	Zuckerman Institute, Columbia University, New York, NY, USA
Inês	C	Laranjeira	Champalimaud Center for the Unknown, Lisboa, Portugal
Peter		Latham	Gatsby Computational Neuroscience Unit, University College London, London, UK
Petrina		Lau	Wolfson Institute of Biomedical Research, University College London, London, United Kingdom
Hyun	D	Lee	Zuckerman Institute, Columbia University, New York, NY, USA
Ari		Liu	Department of Brain and Cognitive Sciences, Massachusetts Institute of Technology, Cambridge, MA, USA
Zachary	F	Mainen	Champalimaud Center for the Unknown, Lisboa, Portugal
Amalia		Makri-Cottington	Wolfson Institute of Biomedical Research, University College London, London, United Kingdom
Hernando		Martinez-Vergara	Sainsbury-Wellcome Centre, University College London, London, UK
Brenna		McMannon	Princeton Neuroscience Institute, Princeton University, Princeton, NJ, USA
Isaiah		McRoberts	Center for Neural Science, New York University, New York, NY, USA
Guido	T	Meijer	Champalimaud Center for the Unknown, Lisboa, Portugal
Maxwell		Melin	Department of Neurobiology, University of California, Los Angeles, CA, USA
Leenoy		Meshulam	Center for Computational Neuroscience, University of Washington, Seattle, WA, USA
Kim		Miller	Department of Biological Structure, University of Washington, Seattle, WA, USA
Nathaniel	J	Miska	Sainsbury-Wellcome Centre, University College London, London, UK
Catalin		Mitelut	Zuckerman Institute, Columbia University, New York, NY, USA
Zeinab		Mohammadi	Princeton Neuroscience Institute, Princeton University, Princeton, NJ, USA

Thomas		Mrsic-Flogel	Sainsbury-Wellcome Centre, University College London, London, UK
Masayoshi		Murakami	Department of Physiology, University of Yamanashi, Kofu, Yamanashi, Japan
Jean-Paul		Noel	Center for Neural Science, New York University, New York, NY, USA
Kai		Nylund	Department of Biological Structure, University of Washington, Seattle, WA, USA
Farideh		Oloomi	Max Planck Institute for Biological Cybernetics, Tübingen, Germany
Alejandro		Pan Vazquez	Princeton Neuroscience Institute, Princeton University, Princeton, NJ, USA
Liam		Paninski	Zuckerman Institute, Columbia University, New York, NY, USA
Sabrina		Perrenoud	Wolfson Institute of Biomedical Research, University College London, London, United Kingdom
Alberto		Pezzotta	Gatsby Computational Neuroscience Unit, University College London, London, UK
Samuel		Picard	Institute of Neurology, University College London, London, UK
Jonathan	W	Pillow	Princeton Neuroscience Institute, Princeton University, Princeton, NJ, USA
Alexandre		Pouget	Department of Basic Neuroscience, University of Geneva, Geneva, Switzerland
Carolina		Quadrado	Institute of Neurology, University College London, London, UK
Pranav		Rai	Champalimaud Center for the Unknown, Lisboa, Portugal
Georg		Raiser	Champalimaud Center for the Unknown, Lisboa, Portugal
Florian		Rau	Champalimaud Center for the Unknown, Lisboa, Portugal
Cyrille		Rossant	Institute of Neurology, University College London, London, UK
Noam		Roth	Department of Biological Structure, University of Washington, Seattle, WA, USA
Nicholas	A	Roy	Princeton Neuroscience Institute, Princeton University, Princeton, NJ, USA
Kamron		Saniee	Zuckerman Institute, Columbia University, New York, NY, USA
Rylan		Schaeffer	Department of Brain and Cognitive Sciences, Massachusetts Institute of Technology, Cambridge, MA, USA
Michael	M	Schartner	Champalimaud Center for the Unknown, Lisboa, Portugal
Yanliang		Shi	Princeton Neuroscience Institute, Princeton University, Princeton, NJ, USA
Karolina	Z	Socha	Institute of Ophthalmology, University College London, London, United Kingdom
Cristian		Soitu	Cold Spring Harbor Laboratory, Cold Spring Harbor, NY, USA
Nicholas	A	Steinmetz	Department of Biological Structure, University of Washington, Seattle, WA, USA

Karel		Svoboda	The Allen Institute for Neural Dynamics, Seattle, WA, USA
Marsa		Taheri	University of California, Los Angeles
Charline		Tessereau	Max Planck Institute for Biological Cybernetics, Tübingen, Germany
Matthew		Tucker	Department of Biological Structure, University of Washington, Seattle, WA, USA
Anne	E	Urai	Cognitive Psychology Unit, Institute of Psychology and Leiden Institute for Brain and Cognition, Leiden University, Leiden, Netherlands
Erdem		Varol	Zuckerman Institute, Columbia University, New York, NY, USA
Shuqi		Wang	Zuckerman Institute, Columbia University, New York, NY, USA
Miles	J	Wells	Institute of Neurology, University College London, London, UK
Steven	J	West	Sainsbury-Wellcome Centre, University College London, London, UK
Matthew	R	Whiteway	Zuckerman Institute, Columbia University, New York, NY, USA
Charles		Windolf	Zuckerman Institute, Columbia University, New York, NY, USA
Olivier		Winter	Champalimaud Center for the Unknown, Lisboa, Portugal
Ilana		Witten	Princeton Neuroscience Institute, Princeton University, Princeton, NJ, USA
Lauren	E	Wool	Institute of Neurology, University College London, London, UK
Zekai		Xu	Gatsby Computational Neuroscience Unit, University College London, London, UK
Kenneth		Yang	Department of Biological Structure, University of Washington, Seattle, WA, USA
Yaxuan		Yang	Sainsbury-Wellcome Centre, University College London, London, UK
Han		Yu	Zuckerman Institute, Columbia University, New York, NY, USA
Anthony	M	Zador	Cold Spring Harbor Laboratory, Cold Spring Harbor, NY, USA
Yizi		Zhang	Zuckerman Institute, Columbia University, New York, NY, USA
