# Supplementary material for: Brain-wide representations of prior information in mouse decision-making
Source: Nature. 2025 Sep 3;645(8079):192–200. doi: 10.1038/s41586-025-09226-1 (PMC12408363; doi:10.1038/s41586-025-09226-1)
Supplement: Supplementary file 2 — Reporting Summary [file 41586_2025_9226_MOESM2_ESM.pdf]

nature portfolio

Corresponding author(s): Charles Findling

Last updated by author(s): Apr 25, 2025

Reporting Summary

Nature Portfolio wishes to improve the reproducibility of the work that we publish. This form provides structure for consistency and transparency in reporting. For further information on Nature Portfolio policies, see our Editorial Policies and the Editorial Policy Checklist.

Statistics

For all statistical analyses, confirm that the following items are present in the figure legend, table legend, main text, or Methods section.

n/a	Confirmed	
☐	☒	The exact sample size (n) for each experimental group/condition, given as a discrete number and unit of measurement
☐	☒	A statement on whether measurements were taken from distinct samples or whether the same sample was measured repeatedly
☐	☒	The statistical test(s) used AND whether they are one- or two-sided<br>*Only common tests should be described solely by name; describe more complex techniques in the Methods section.*
☐	☒	A description of all covariates tested
☐	☒	A description of any assumptions or corrections, such as tests of normality and adjustment for multiple comparisons
☐	☒	A full description of the statistical parameters including central tendency (e.g. means) or other basic estimates (e.g. regression coefficient) AND variation (e.g. standard deviation) or associated estimates of uncertainty (e.g. confidence intervals)
☐	☒	For null hypothesis testing, the test statistic (e.g. F, t, r) with confidence intervals, effect sizes, degrees of freedom and P value noted<br>*Give P values as exact values whenever suitable.*
☐	☒	For Bayesian analysis, information on the choice of priors and Markov chain Monte Carlo settings
☐	☒	For hierarchical and complex designs, identification of the appropriate level for tests and full reporting of outcomes
☐	☒	Estimates of effect sizes (e.g. Cohen's d, Pearson's r), indicating how they were calculated

Our web collection on statistics for biologists contains articles on many of the points above.

Software and code

Policy information about availability of computer code

Data collection	please see https://int-brain-lab.github.io/iblenv/notebooks_external/data_structure.html
Data analysis	all our code is available at https://github.com/int-brain-lab/prior-localization<br>Requirements and versions are:<br>scikit-learn==1.5.1<br>psychofit @ git+https://github.com/cortex-lab/psychofit.git<br>behavior_models @ git+https://github.com/int-brain-lab/behavior_models.git<br>brainwidemap @ git+https://github.com/int-brain-lab/paper-brain-wide-map.git

For manuscripts utilizing custom algorithms or software that are central to the research but not yet described in published literature, software must be made available to editors and reviewers. We strongly encourage code deposition in a community repository (e.g. GitHub). See the Nature Portfolio guidelines for submitting code & software for further information.

Data

Policy information about availability of data

All manuscripts must include a data availability statement. This statement should provide the following information, where applicable:

- Accession codes, unique identifiers, or web links for publicly available datasets
- A description of any restrictions on data availability
- For clinical datasets or third party data, please ensure that the statement adheres to our policy

The electrophysiology data for this paper are available via http://viz.internationalbrainlab.org and https://int-brain-lab.github.io/iblenv/notebooks_external/data_release_brainwidemap.html
The widefield and pupil tracking data are also available through the public IBL database (https://openalyx.internationalbrainlab.org) and can be accessed via the ONE API using the tag "2023_Q3_Findling_Hubert_et_al" as described here https://int-brain-lab.github.io/ONE/notebooks/one_search/one_search.html#Searching-data-with-a-release-tag.
The Swanson flat map can be found at https://int-brain-lab.github.io/iblenv/notebooks_external/atlas_swanson_flatmap.html

Research involving human participants, their data, or biological material

Policy information about studies with human participants or human data. See also policy information about sex, gender (identity/presentation), and sexual orientation and race, ethnicity and racism.

Reporting on sex and gender	N/A
Reporting on race, ethnicity, or other socially relevant groupings	N/A
Population characteristics	N/A
Recruitment	N/A
Ethics oversight	N/A

Note that full information on the approval of the study protocol must also be provided in the manuscript.

Field-specific reporting

Please select the one below that is the best fit for your research. If you are not sure, read the appropriate sections before making your selection.

☒ Life sciences ☐ Behavioural & social sciences ☐ Ecological, evolutionary & environmental sciences

For a reference copy of the document with all sections, see nature.com/documents/nr-reporting-summary-flat.pdf

Life sciences study design

All studies must disclose on these points even when the disclosure is negative.

Sample size	No statistical methods were used to predetermine sample sizes. For electrophysiology, data were collected from 699 Neuropixels probe insertions across 459 sessions in 139 mice, with 414 sessions meeting inclusion criteria. For widefield calcium imaging, 51 sessions from 6 mice were included. Regions were analyzed if at least 5 well-isolated units (Ephys) or pixels (WFI) passed quality control. These sample sizes ensured sufficient statistical power for decoding analyses and robust brain-wide coverage, as supported by estimates indicating ~10 recordings per region are sufficient (see fig S5e).
Data exclusions	Pre-established exclusion criteria were applied to ensure data quality. Trials were excluded if mice did not respond, or if reaction times were <80ms or >2s. Sessions with <250 included trials were excluded (41 Ephys, 1 WFI). For electrophysiology, only neurons passing strict quality control (amplitude >50µV, noise cut-off <20, and no refractory period violations) were included. Regions required ≥5 QC-passed units (Ephys) or pixels (WFI) to be analyzed. These criteria were defined prior to analysis and are detailed in the Methods.
Replication	The main behavioral and neural analyses were replicated across two independent recording modalities—Neuropixels electrophysiology (699 insertions across 459 sessions in 139 mice) and widefield calcium imaging (51 sessions in 6 mice). Behavioral effects and neural decoding of the prior were consistent across both modalities.
Randomization	Randomization into experimental groups was not applicable, as all mice were trained on the same task and underwent the same recording procedures. To assess significance in decoding neural representations, we employed a pseudo-session resampling procedure to construct null distributions, as detailed in the Methods.
Blinding	Blinding was not performed because all mice were trained using identical protocols and recorded using standardized procedures. There were no experimental groups or treatment conditions to blind against.

Reporting for specific materials, systems and methods

We require information from authors about some types of materials, experimental systems and methods used in many studies. Here, indicate whether each material, system or method listed is relevant to your study. If you are not sure if a list item applies to your research, read the appropriate section before selecting a response.

Materials & experimental systems

n/a	Involved in the study
☒	☐ Antibodies
☒	☐ Eukaryotic cell lines
☒	☐ Palaeontology and archaeology
☐	☒ Animals and other organisms
☒	☐ Clinical data
☒	☐ Dual use research of concern
☒	☐ Plants

Methods

n/a	Involved in the study
☒	☐ ChIP-seq
☒	☐ Flow cytometry
☒	☐ MRI-based neuroimaging

Animals and other research organisms

Policy information about studies involving animals; ARRIVE guidelines recommended for reporting animal research, and Sex and Gender in Research

Laboratory animals	we used C57BL/6 laboratory mice
Wild animals	No wild animals were used in this study
Reporting on sex	both sexes were used; and are reported
Field-collected samples	No field-collected samples were used in this study
Ethics oversight	All experimental procedures involving animals were conducted in accordance with local laws and approved by the relevant institutional ethics committees. Approvals were granted by the Animal Welfare Ethical Review Body of University College London, under licences P1DB285D8, PCC4A4ECE, and PD867676F, issued by the UK Home Office. Experiments conducted at Princeton University were approved under licence 1876-20 by the Institutional Animal Care and Use Committee (IACUC). At Cold Spring Harbor Laboratory, approvals were granted under licences 1411117 and 19.5 by the IACUC. The University of California at Los Angeles granted approval through IACUC licence 2020-121-TR-001. Additional approvals were obtained from the University Animal Welfare Committee of New York University (licence 18-1502); the IACUC at the University of Washington (licence 4461-01); the IACUC at the University of California, Berkeley (licence AUP-2016-06-8860-1); and the Portuguese Veterinary General Board (DGAV) for experiments conducted at the Champalimaud Foundation (licence 0421/0000/0000/2019).

Note that full information on the approval of the study protocol must also be provided in the manuscript.

Plants

Seed stocks	N/A
Novel plant genotypes	N/A
Authentication	N/A
